# Coagulant plus ballast technique provides a rapid mitigation of cyanobacterial nuisance

**DOI:** 10.1371/journal.pone.0178976

**Published:** 2017-06-09

**Authors:** Natalia P. Noyma, Leonardo de Magalhães, Marcela Miranda, Maíra Mucci, Frank van Oosterhout, Vera L. M. Huszar, Marcelo M. Marinho, Eduardo R. A. Lima, Miquel Lürling

**Affiliations:** 1 Laboratory of Ecology and Physiology of Phytoplankton, Department of Plant Biology, University of Rio de Janeiro State, Rio de Janeiro, Brazil; 2 Aquatic Ecology & Water Quality Management Group, Department of Environmental Sciences, Wageningen University, Wageningen, The Netherlands; 3 Museu Nacional, Federal University of Rio de Janeiro, Rio de Janeiro, Brazil; 4 Chemistry Institute, University of Rio de Janeiro State, Rio de Janeiro, Rio de Janeiro, Brasil; 5 Department of Aquatic Ecology, Netherlands Institute of Ecology (NIOO-KNAW), Wageningen, The Netherlands; Natural Environment Research Council, UNITED KINGDOM

## Abstract

Cyanobacteria blooms are a risk to environmental health and public safety due to the potent toxins certain cyanobacteria can produce. These nuisance organisms can be removed from water bodies by biomass flocculation and sedimentation. Here, we studied the efficacy of combinations of a low dose coagulant (poly-aluminium chloride—PAC—or chitosan) with different ballast compounds (red soil, bauxite, gravel, aluminium modified zeolite and lanthanum modified bentonite) to remove cyanobacterial biomass from water collected in Funil Reservoir (Brazil). We tested the effect of different cyanobacterial biomass concentrations on removal efficiency. We also examined if zeta potential was altered by treatments. Addition of low doses of PAC and chitosan (1–8 mg Al L^-1^) to the cyanobacterial suspensions caused flock formation, but did not settle the cyanobacteria. When those low dose coagulants were combined with ballast, effective settling in a dose-dependent way up to 99.7% removal of the flocks could be achieved without any effect on the zeta potential and thus without potential membrane damage. Removal efficacy was influenced by the cyanobacterial biomass and at higher biomass more ballast was needed to achieve good removal. The combined coagulant-ballast technique provides a promising alternative to algaecides in lakes, ponds and reservoirs.

## Introduction

Cyanobacteria blooms are a threat to environmental health and public safety as many cyanobacteria produce a variety of potent toxins that have been implicated in human and animal illness and death in over fifty countries worldwide [1–3]. Blooms of cyanobacteria may compromise important ecosystems services and come with substantial costs for water users worldwide through increased treatment costs, and loss of recreational and aquaculture revenue [4, 5]. Ongoing urbanisation and global warming are expected to aggravate the intensity, frequency and duration of cyanobacterial blooms [6–8]. Therefore, water authorities are in dire need for an array of both curative and preventive measures to counteract cyanobacteria proliferation and control nuisance blooms.

It is generally accepted that cyanobacteria proliferation is strongly nutrient driven [7, 9, 10]. Consequently, strong reduction of external nutrient inputs to the receiving water bodies is the most obvious first step in controlling cyanobacterial proliferations [11, 12]. Although such catchment management is the most desirable approach, ecological responses following successful external nutrient load reduction can take decades to centuries [13]. Moreover, economically feasible reductions of external nutrient loading are not always possible, leaving curative, or end-of-pipe effect-oriented, measures as the most suited nuisance control in those open systems [14]. The effects of curative measures are generally short lasting and will need to be repeated regularly, which means the application of these measures should be fast, easy and cheap. In addition, such interventions should be safe; environmentally safe management strategies should not only mitigate blooms, but also prevent toxin release from the cyanobacteria [15]. Common curative interventions are based on using algaecides that may efficiently eliminate blooms, but also induce cyanobacterial cell lysis and consequently release of intracellular toxins [14, 16]. Recently, hydrogen peroxide has been proposed as an environmental friendly alternative to metal based algaecides [17], but hydrogen peroxide induces cyanobacterial cell lysis after which increased dissolved cyanotoxin (microcystin) concentrations have been found [18, 19]. A seemingly promising alternative to algaecides is to flock and sink the cyanobacteria out of the water column while remaining as intact cells [20, 21], where after the cyanobacteria and their toxins can be degraded on the sediment [22–26].

As ballast compounds, solid phase phosphate sorbents could be used, such as lanthanum modified bentonite, which has the advantage to block sediment phosphate release [20, 27–29]. These compounds are particularly well suited for water bodies with a relatively large internal P-load compared to external P-load and good results have been obtained with lanthanum modified bentonite combined with a coagulant [20, 29]. However, in waters with strong ongoing external P-load the use of relatively expensive modified P adsorbents is not recommended. In those cases that the external load reduction is not feasible to be implanted, the use of local soils, clays or waste products can be a fast, cheap and ease of handling alternative to reduces the internal P-loading [30], especially in developing countries. In developing countries, sewerage coverage and sewage treatment of domestic waste water is poor which requires huge investments to manage these point sources [31]. In these cases, the external load control is unattainable [32] leading to a do-nothing approach for the decades to come in those countries. Evidently, such attitude is undesirable and also unnecessary because the “Flock & Lock” principle [20] can be extended easily with non-, or less P—adsorbing compounds to a “Flock & Sink” approach as natural soils and clays modified with flocculants can effectively remove cyanobacteria from the water column [21, 27, 31, 33–35]. However, more information is needed on the suitability of such local products as ballast compounds -needed in conjunction with a coagulant- in settling cyanobacteria blooms.

Some factors should be taken into consideration for a successful “Flock & Sink” approach. First, the low dose coagulant should be effective in flocking the cells without causing damage. Hereto, it is important that the zeta-potential is not elevated too much to prevent loss of membrane integrity and subsequent leakage of cell constituents [36, 37]. Second, the ballast compounds themselves should be tested on their cyanobacteria settling capacity and it has been shown that a combination with a low dose of a coagulant is needed for effective flocking and the ballast for settling positively buoyant cyanobacteria [21, 27, 38, 39]. Third, it is expected that the nature of the ballast is of minor influence on removal efficacy and that world-wide occurring materials like red soil, bauxite and gravel make good candidates. Finally, since cyanobacterial blooms may vary considerably in density, it can be expected that bloom density will determine the amount of ballast needed to settle the cyanobacteria effectively out of the water column. This last aspect has, however, not been addressed in the literature so far.

## Materials and methods

### Sampling

Water samples were taken monthly from September to December 2015 from the Funil Reservoir which is located in the southern part of Rio de Janeiro State, Brazil (22°30’S, 44°45’W, altitude 440 m). No specific permissions were required for taken water samples from Funil reservoir under Brazilian law.

The reservoir receives water from the Paraíba do Sul River, it has a catchment area of 16,800 km^2^, a surface area of 40 km^2^, a mean and maximum depth of 22 and 70 m, respectively, a mean total water volume of 890 × 10^6^ m^3^, which may vary considerably depending on climate conditions and on the water use for energy, and a residence time of 20 to 80 days [40]. The climate conditions are a wet-warm summer and dry-mild cold winter (Cwa in the Köppen system). Funil is a eutrophic reservoir with median total P concentration > 40 μg L^-1^ [41]. Also during September 2015 sampling there was a heavy bloom, with scums accumulating at the surface (chlorophyll *a* > 4000 μg L^-1^). This bloom was comprised of *Dolichospermum circinalis* (Rabenhorst) Wacklin, Hoffmann & Komárek, and during the others sampling months dominance of *Microcystis aeruginosa* (Kützing) Kützing was observed.

### Chemicals and materials

Local red soil (LRS) was collected from the banks of Funil Reservoir. Bauxite (BAU) was obtained from Mineração Rio do Norte S.A (Pará, Brazil). Gravel (GRA)—commonly available under the local name of *Saibro Roxo* that contains a large amount of feldspar and quartz fragments (<0.5 mm)—was bought in a construction store (Mineração, Juiz de Fora, Brazil). The La modified bentonite Phoslock^®^ (LMB) was obtained from HydroScience (Porto Alegre, Brazil). This LMB was developed by the Australian CSIRO, as dephosphatisation technique aiming at removing SRP from the water and blocking the release of SRP from the sediment [42]. The aluminium modified zeolite Aqual-P (AMZ) was obtained from Blue Pacific Minerals (Tokoroa, New Zealand). AMZ is a modified zeolite which acts as aluminium (Al)-based P-binding agent [43]. The coagulant PAC-AP (poly-aluminium chloride; Al_n_(OH)_m_Cl_3n_-_m_, ρ ≈ 1.37 kg L^-1^, 8.9% Al, 21.0% Cl) was obtained from Pan-Americana (Rio de Janeiro, Brazil), whereas chitosan made of shrimp shells was obtained from Polymar Ciência e Nutrição S/A (Ceará, Brazil). The chitosan was acidified with a 1% hydrochloric acid solution prior to use and diluted to a stock of 1 g L^-1^.

### Flock & Sink assays

Positively buoyant cyanobacteria from Funil Reservoir tended to aggregate rapidly at the surface of the water column in test tubes. In the Flock & Sink assays different compounds (described below) were tested on their ability to counteract this surface aggregation and to settle the cyanobacteria in the bottom of the tubes. Hereto, aliquots of 50 mL cyanobacteria suspensions from Funil Reservoir were transferred to 75 mL glass tubes (25x200 mm). The initial cyanobacterial chlorophyll-*a* concentration (μg L^-1^) and Photosystem II (PSII) efficiency were determined using a PHYTO-PAM phytoplankton analyser (Heinz Walz GmbH, Effeltrich, Germany). The suspensions were treated with the designated compound(s) (treatment) or left untreated (controls), mixed and placed in the laboratory at 25°C under stagnant conditions. After one hour 5 mL samples were taken from both the top and the bottom of the tubes in which the chlorophyll-*a* concentrations and PSII efficiencies were measured. PSII efficiency value can be used as an indicative of physiological stress in algae [44, 45].

The pH was measured in the tubes using a Lutron (model pH-212) pH meter. The zeta potential (mV) was measured using a Nanoparticle Analyser (SZ-100, Horiba Scientific). The sample was injected into a disposable cell and a measurement of the particle electrophoretic mobility resulted in the calculated zeta potential provided by the equipment.

#### PAC, chitosan and local red soil (LRS)

Low doses of the coagulants PAC (0, 1, 2, 4 and 8 mg Al L^-1^) and chitosan (0, 1, 2, 4 and 8 mg L^-1^) were tested on their flocculation and ability to settle the positively buoyant cyanobacteria. In addition, the ballast compound LRS (0, 25, 50, 100, 200 and 400 mg L^-1^) was tested alone, in combination with PAC (2 mg Al L^-1^) and in combination with chitosan (2 mg L^-1^). The concentration of both coagulants was chosen based on zeta potential measurements (Table 1). A slurry of the LRS was made with water from the test tube, where after the slurry was sprayed on the water surface in the tube. When combined with a coagulant, the PAC or chitosan was added immediately after addition of the LRS and the tubes were mixed using a glass rod. The initial chlorophyll-*a* concentration of the suspensions was 145 (± 9) μg L^-1^ with cells having a PSII-efficiency of 0.31 (± 0.01). Chlorophyll-*a* measurements are included as a measure of biomass, while PSII-efficiency is included to evaluate the physiological condition of the cells. In healthy cells the efficiency with which light energy is utilized is higher than when cells are under stress.

**Table 1:** Chlorophyll-*a* concentration (micrograms per liter), Photosystem II efficiency (PSII), pH, zeta potential (mV) and percentage of cyanobacterial biomass removal in the top 5 mL and bottom 5 mL of 50 mL cyanobacteria suspensions incubated for one hour in a range of PAC (poly-alumnium chloride), chitosan and local red soil (LRS) series concentrations.

PAC (mg L^-1^)	Chlorophyll-*a* (μg L^-1^)		PSII-efficiency (-)		pH (-)	Zeta potential (mV)		Removal (%)
	top	bottom	top	bottom		top	bottom	
0	1129	78	0.29	0.41	6.3	-38	-34	--
1	1323	57	0.29	0.42	6.3	-45.6	-34.8	-17.2
2	1375	45	0.43	0.42	6.2	-43.1	-34	-21.7
4	971	28	0.39	0.41	5.9	-24.1	-19.9	14.0
8	1575	30	0.27	0.41	5.7	0.1	10.7	-39.4
Chitosan (mg L^-1^)	Chlorophyll-*a* (μg L^-1^)		PSII-efficiency (-)		pH (-)	Zeta potential (mV)		Removal (%)
	top	bottom	top	bottom		top	bottom	
0	1089	80	0.32	0.4	6.5	-37.9	-35.3	--
1	1167	65	0.32	0.41	6.5	-41.3	nd	-7.2
2	1073	47	0.31	0.42	6.4	-39	-36.6	1.4
4	814	43	0.36	0.39	6.2	-2.3	-34.6	25.2
8	1052	65	0.33	0.35	5.9	-6.1	-15.7	3.3
LRS (mg L^-1^)	Chlorophyll-*a* (μg L^-1^)		PSII-efficiency (-)		pH (-)	Zeta potential (mV)		Removal (%)
	top	bottom	top	bottom		top	bottom	
0	1145	73	0.32	0.38	6.5	-44.1	-36.5	--
25	1023	93	0.33	0.42	6.5	-43.7	-34.4	10.6
50	1249	141	0.33	0.38	6.5	-35.8	-33.5	-9.1
100	1058	213	0.33	0.37	6.5	-37	-34.5	7.6
200	1054	375	0.33	0.4	6.5	-39.5	-36.9	7.9
400	802	562	0.35	0.36	6.5	-34.9	-38.4	29.9

#### Different ballast compounds

Five different ballast compounds—aluminium modified zeolite (AMZ), bauxite (BAU), gravel (GRA), lanthanum modified bentonite (LMB) and local red soil (LRS)—were examined on their ability to settle the positively buoyant cyanobacteria. LMB and AMZ have strong P binding properties and are representing “Flock & Lock” ballast, while BAU, GRA and LRS are representing the ballast in “Flock & Sink”. Each of the compounds was tested at five doses (25, 50, 100, 200 and 400 mg L^-1^), while one test tube for each compound series remained untreated (controls). The ballast compounds were added by making a slurry with some water from the designated test tubes and spraying it on the surface of the tubes and gently mixed using a glass rod. These experiments were run in triplicate. After one hour, water samples were taken from the top of the test tubes and the remaining cyanobacterial biomass (as chlorophyll-*a*) was determined.

Here after, the experiment was repeated with cyanobacterial suspensions exposed to the same ballast compounds and doses in presence of a low dose coagulant (PAC or chitosan) aimed to aid the settling process. The coagulants were PAC that was used in a fixed dose of 2 mg Al L^-1^ and chitosan dosed at 2 mg L^-1^. Each series was run in triplicate and the chlorophyll-*a* concentrations remaining in the top of the test tubes after treatment were statistically compared running a two-way ANOVA with ballast compound and ballast dose as the fixed factors. Hence, we tested the scum removal in this analysis. Exponential decay curves were fit to chlorophyll-*a* data from the top of the tubes from each series treatment over different ballast concentrations and the difference between the calculated decay constants of PAC or chitosan additions were compared running a t-test. All the statistical analyses were performed in the program SigmaPlot (version 13).

#### Cyanobacterial biomass

The amount of ballast (LRS) needed to settle a different concentration of positively buoyant cyanobacteria was examined. Surface accumulated material from Funil Reservoir was diluted with filtered surface water to obtain six different cyanobacterial concentrations: 30, 55, 95, 187, 361 and 651 μg chlorophyll-*a* L^-1^. For each cyanobacterial concentration six test tubes were filled with 50 mL suspension that were subsequently treated with different doses LRS (25, 50, 100, 200 and 400 mg L^-1^) in combination with PAC (2 mg Al L^-1^). One tube per cyanobacterial concentration was left untreated (control). Immediately after adding the slurry of LRS, PAC was added and the tubes were mixed using a glass rod. The slopes and intercepts of regression lines on natural log transformed chlorophyll-*a* concentrations in the top of the test tubes after treatments were statistically compared running a Parallel Lines Analysis (SigmaPlot, version 13).

## Results

### PAC, chitosan and LRS

As compared to the controls, the addition of PAC (1–8 mg L^-1^) resulted in a 20% higher biomass (chlorophyll-*a*) accumulating in the top of the tubes (Table 1). The chlorophyll-*a* concentrations at the bottom of the test tubes were low (on average 48 μg L^-1^) compared to those in the top of the tubes (on average 1274 μg L^-1^) (Table 1). The pH declined slightly with increasing PAC dose (Table 1). Likewise, for chitosan at all doses tested (1–8 mg L^-1^) cyanobacteria accumulated at the water surface (on average 1039 μg L^-1^), while the chlorophyll-*a* concentrations in the bottom of the test tubes were much lower (on average 60 μg L^-1^). The flocks formed in the treatments with chitosan were visually smaller than those in the PAC treatments; only at the highest chitosan dose the pH was slightly lower compared to the other chitosan treatments (Table 1). In all LRS doses cyanobacteria accumulated at the water surface, albeit 30% less in the highest dose (400 mg L^-1^) compared to the control. In the bottom, there was a gradual increase of cyanobacterial biomass with LRS dose (Table 1). This increase (Cyanobottom) was linearly related to the LRS dose applied: Cyanobottom = 80.384 + 1.259 × LRS (r^2^ = 0.985, P < 0.05). LRS had no effect on pH (Table 1). There was no obvious effect on PSII-efficiency observed in any of the treatments (Table 1).

**Fig 1:**
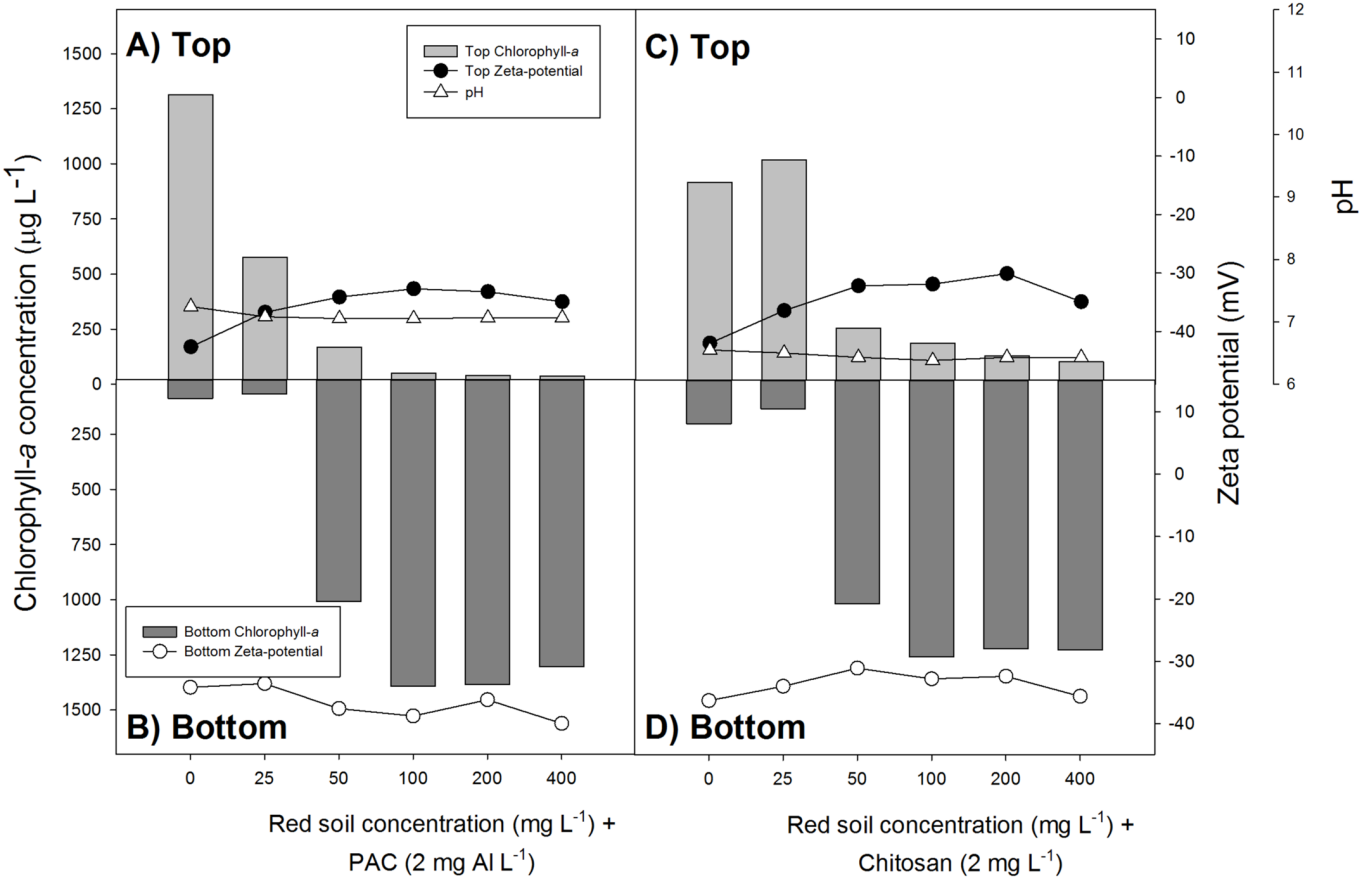
Chlorophyll-*a* concentrations (micrograms per liter) in the top 5 mL (top light gray bars) and bottom 5 mL (lower dark gray bars) of 50 mL cyanobacteria suspensions incubated for one hour with different concentrations of the red soil and the flocculants PAC (poly-aluminium chloride, 2 mg Al L^-1^; panel A and B) or chitosan (2 mg L^-1^; panel C and D). Also included are the pH values (open triangles) of the suspensions and zeta potential in top (filled circles) and bottom (open circles).

When LRS was combined with either PAC (2 mg Al L^-1^) or chitosan (2 mg L^-1^) at doses of 50 mg L^-1^ and higher, the cyanobacteria were settled to the bottom of the test tubes (Fig 1, S1 Dataset). Consequently, there was less biomass accumulation at the water surface; compared to the controls, chlorophyll-*a* concentrations in the chitosan-LRS (50–400 mg L^-1^) treatments were 72 to 89% lower, while in the PAC-LRS treatments this was 87 to 97% (Fig 1).

PSII-efficiencies were not obviously changed in the treatments and were, on average, 0.38 (± 0.03) in the top of the tubes in the chitosan-LRS series, 0.41 (± 0.04) in the bottom of the tubes in that series, 0.37 (± 0.04) in the top of the tubes in the PAC-LRS series and 0.39 (± 0.02) in the bottom. The pH was 6.45 (± 0.06) in the chitosan-LRS series and 6.28 (± 0.09) in the PAC-LRS series (Fig 1).

#### Zeta potential

**Fig 2:**
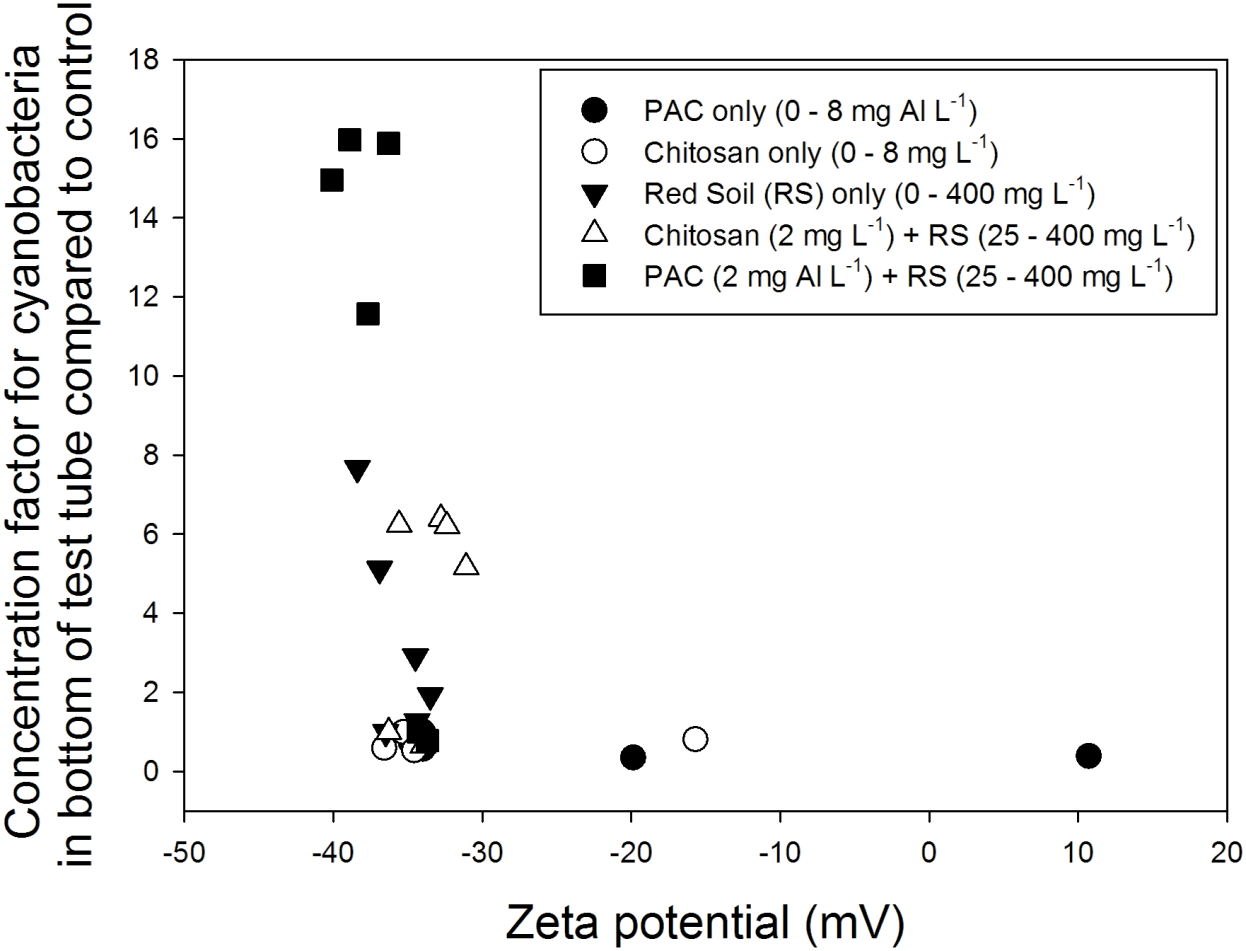
Variation of the zeta potential values (mV) of concentrations factor for cyanobacteria in the bottom of test tubes compared to control in PAC (poly-alumnium chloride), chitosan and local red soil (LRS) series concentrations and different concentrations of the red soil and the flocculants PAC (2 mg Al L^-1^;) or chitosan (2 mg L^-1^).

Increasing doses of PAC resulted in less negative zeta potentials and even caused positive values at the highest dose of 8 mg Al L^-1^ (Table 1). Also, higher doses of chitosan led to less negative zeta potentials, while LRS had no effect on the zeta potential of the cyanobacterial suspensions (Table 1). Formation of cyanobacterial flocks already occurred in low dose PAC and chitosan treatments, where no change in zeta potentials was observed (Table 1). When those low dose coagulants were combined with a ballast, effective settling of the flocks could be achieved without any effect on the zeta potentials (Fig 1). That effective settling of cyanobacteria-ballast-coagulant flocks can be achieved without modification of the zeta potential is illustrated in Fig 2, where the cyanobacterial accumulation in the bottom of the test tubes compared to the controls is plotted against the measured zeta potentials (Fig 2). Due to the positive buoyancy of the cyanobacteria, the formed flocks in the absence of a ballast (LRS) migrated rapidly to the surface of the test tubes instead of being sedimented.

### Different ballast compounds

**Fig 3:**
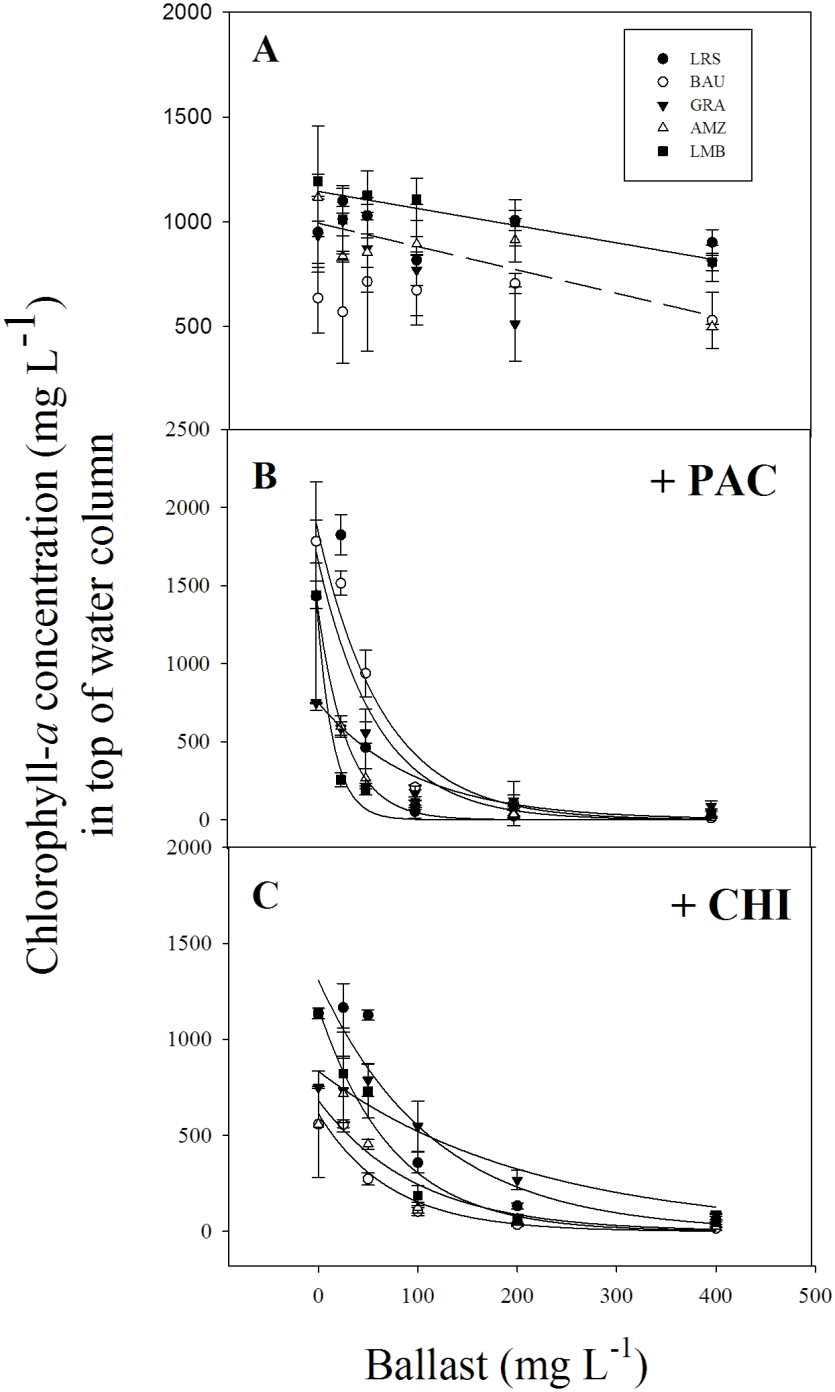
Chlorophyll-*a* concentrations (micrograms per liter) in the top 5 mL of 50 mL cyanobacteria suspensions incubated for one hour with different concentrations of the local red soil (LRS), bauxite (BAU), gravel (GRA), aluminum modified zeolite (AMZ), lanthanum modified bentonite (LMB) solely (A) or with combination the flocculants PAC (poly-aluminium chloride, 2 mg Al L^-1^; B) or chitosan (2 mg L^-1^; C). Statistically significantly exponential decay curves were fit to chl-*a* data over different ballast concentrations. Error bars indicate one standard deviation (*n* = 3).

Considering solely the ballast application (Fig 3A, S1 Dataset), only the modified clays (AMZ and LMB) were able to sediment 56% and 33% of the cyanobacterial biomass at the highest dose (400 mg L^-1^) of AMZ and LMB, respectively. In combination with a low dose PAC all ballast compounds reduced the cyanobacterial biomass from the water surface in a dose-dependent way (Fig 3B, S1 Dataset). A two-way ANOVA showed a significant ballast type effect (F_5,60_ = 53.15; P<0.001), a significant dose effect (F_4,60_ = 146.38; P<0.001) and a significant ballast x dose interaction (F_20,60_ = 14.73; P<0.001). The interaction effect means that the course of the decline in cyanobacterial biomass with ballast dose depends on the ballast used. At the lowest dose (25 mg L^-1^) removal of cyanobacteria from the top water layer was significantly less with LRS and BAU compared to GRA, AMZ and LMB (Tukey test; *p* < 0.05; Fig 3B). With increasing ballast doses the concentrations of remaining cyanobacteria in the top of the tubes converged (Fig 3B) and at the highest ballast dose (400 mg L^-1^) they were equally low (on average 66.9 ± 42.7 μg chlorophyll-*a* L^-1^; Kruskal-Wallis ANOVA on Ranks *H* (4) = 3.033, P = 0.552) Compared to the controls, the highest ballast dose resulted in 88% (GRA) to 99% (AMZ, BAU) less cyanobacteria (Fig 3B). The same pattern was observed for ballasts combined with chitosan (Fig 3C, S1 Dataset). A two-way ANOVA indicated a significant ballast type effect (F_5,60_ = 41.23; P<0.001), a significant dose effect (F_4,60_ = 159.34; P<0.001) and a significant ballast x dose interaction (F_20,60_ = 6.52; P<0.001). Also, here cyanobacteria were depleted stronger from the upper water layers in the tubes with higher doses of ballasts converging to equally low concentrations remaining at the highest ballasts dose of 400 mg L^-1^ (one-way ANOVA: *F*_4,14_ = 2.282; P = 0.132; Fig 3C). At this highest dose, the percentage of cyanobacterial removal compared to the controls varied from 87% (GRA) to 97% (BAU).

The exponential decay constants were significant higher in the treatments with PAC additions compared to Chitosan for LRS (P = 0.024), AMZ (P = 0.0018) and LMB (P < 0.001) (Table 2).

**Table 2:** The exponential decay constants (const), r^2^ and *p* values of the from exponential decay curves fitted to chl-*a* data over different ballast concentrations. Local red soil (LRS), bauxite (BAU), gravel (GRA), aluminum modified zeolite (AMZ), lanthanum modified bentonite (LMB) solely (Ballast) or with combination the flocculants PAC (poly-aluminium chloride, 2 mg Al L^-1^; Ballast+PAC) or chitosan (2 mg L^-1^; Ballast+CHI).

Ballast	Ballast			Ballast +PAC			Ballast +CHI		
	const	r^2^	P	const	r^2^	P	const	r^2^	P
GRA	-0.2417	0.1309	0.481	0.0055	0.6518	0.007	0.0047	0.8555	<0.0001
BAU	-0.215	0.1858	0.393	0.0151	0.9494	<0.0001	0.0087	0.8743	<0.0001
LRS	-0.5584	0.2397	0.324	0.0167	0.6857	0.0077	0.0102	0.7081	0.0039
AMZ	-1.1056	0.6151	0.04	0.0338	0.989	<0.0001	0.0134	0.9195	<0.0001
LMB	-0.8106	0.8028	0.015	0.0603	0.9669	<0.0001	0.0138	0.7958	0.0006

### Cyanobacterial biomass

**Fig 4:**
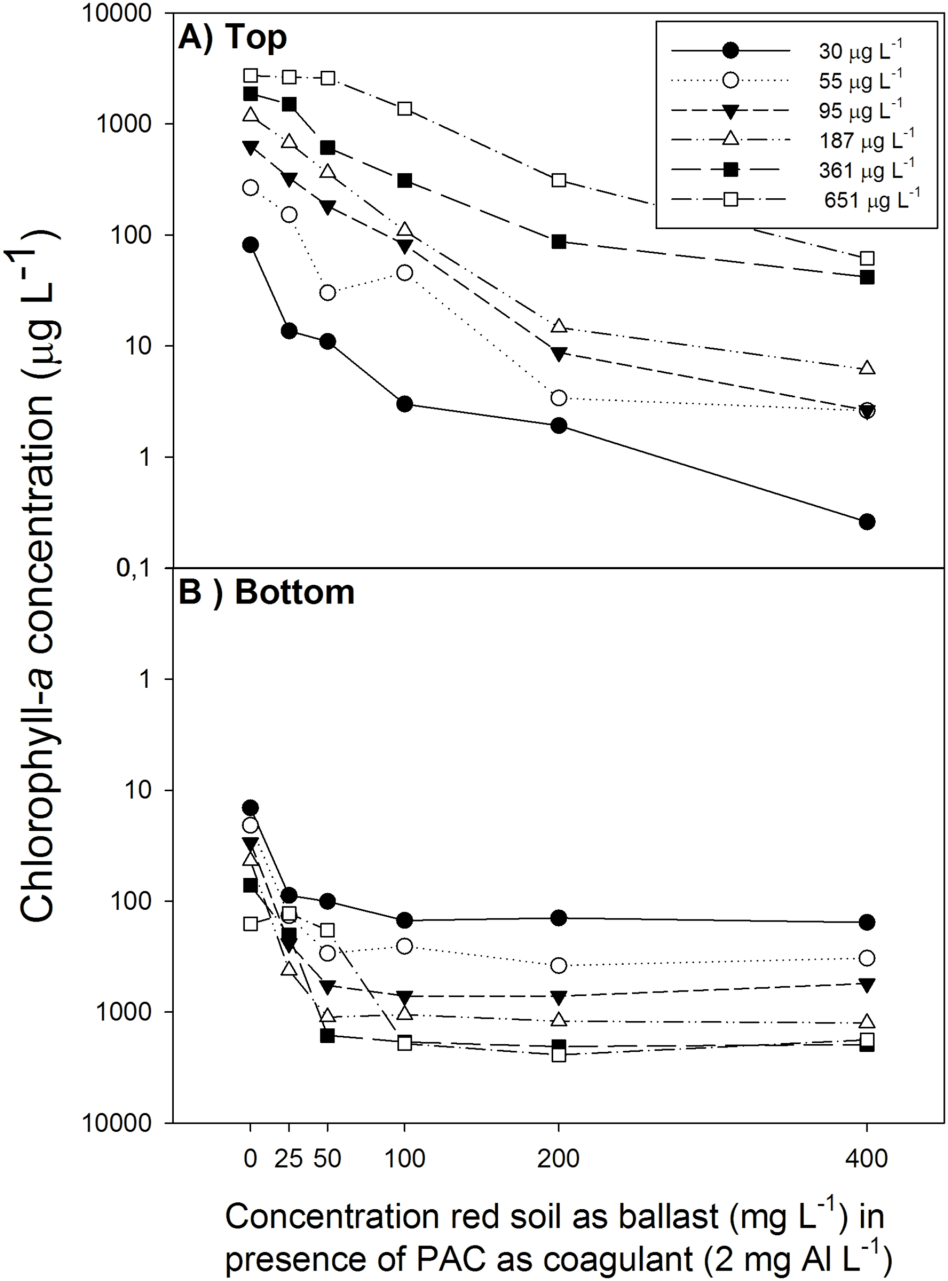
Effect of the initial cyanobacterial biomass variation on the chlorophyll-*a* concentration on 5 mL top (painel A) and 5 mL bottom (painel B) using different local red soil concentration in presence of coagulant PAC (poly-alumnium chloride, 2 mg Al L^-1^).

**Fig 5:**
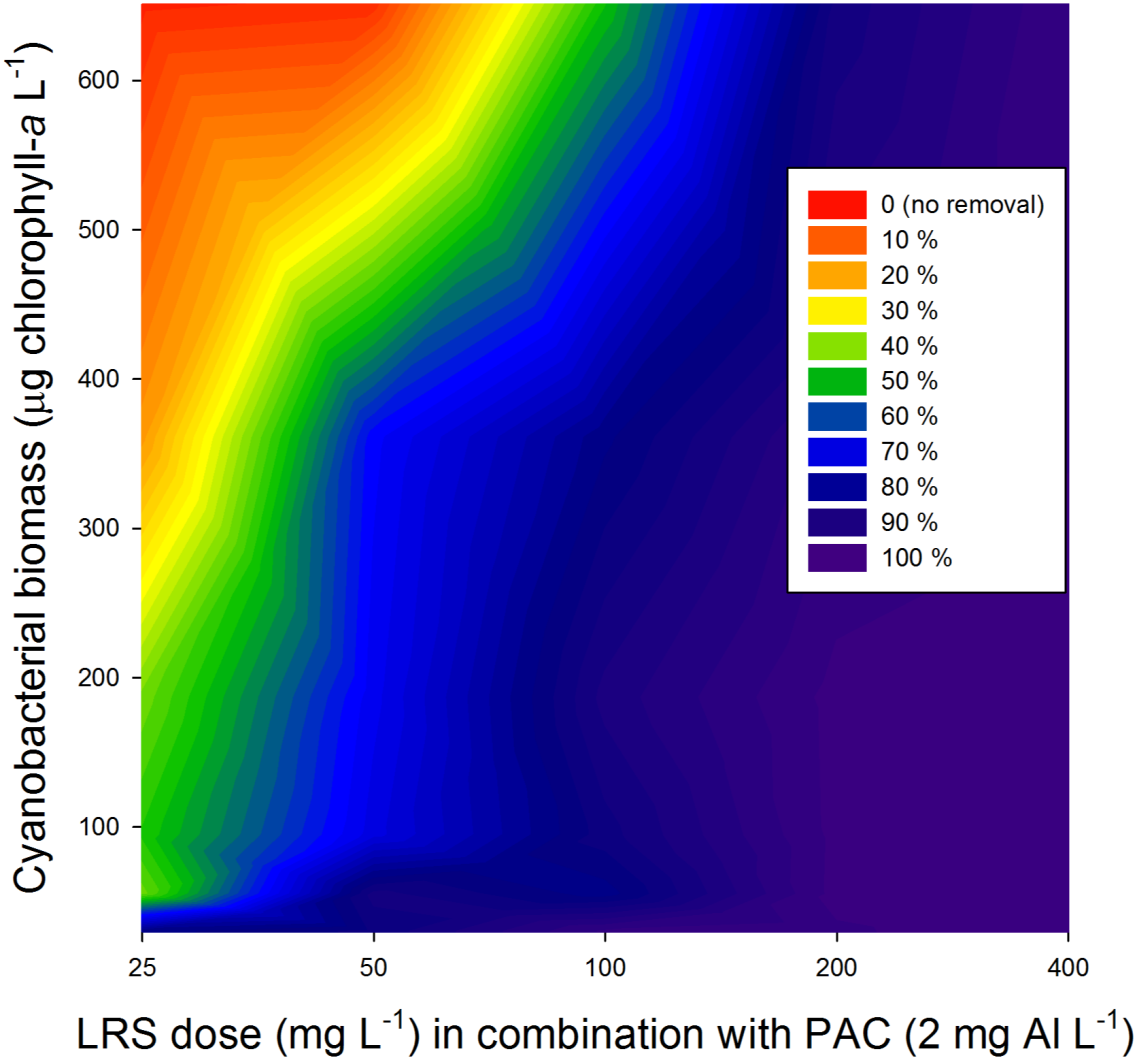
Percentage of cyanobacterial biomass (microgram of chlorophyll-*a* per liter) removal using different local red soil concentration in presence of coagulant PAC (poly-alumnium chloride, 2 mg Al L^-1^).

At all cyanobacterial concentrations tested, a substantial biomass could be sedimented depending on the ballast dose added (Fig 4, S1 Dataset). Cyanobacteria, from the upper water layer, declined with increasing ballast dose and the removed cyanobacteria accumulated in the bottom of the test tubes (Fig 4). A Parallel Lines Analysis revealed that the removal rates were similar (*F*_5,24_ = 0.66; *P* = 0.660), i.e., the decline of the concentrations remaining in the upper water layer with ballast dose added decreased in a similar way during the one hour test duration (overall slope is -0.0118). Consequently, the effectiveness of removal was influenced by the cyanobacterial biomass (Fig 5). At higher cyanobacterial biomass, more ballast was needed to achieve good removal. For instance, where at 30 μg L^-1^ chlorophyll-*a* 100 mg L^-1^ LRS caused a 96% reduction in the cyanobacteria in the top water layer compared to the control, this was only 50% in 651 μg L^-1^ chlorophyll-*a* (Fig 5). The highest dose of ballast tested (400 mg L^-1^ LRS) yielded removal in low cyanobacterial biomass suspensions (99.7% reduction) as well as high biomass suspensions (97.8% reduction).

## Discussion

Our results show that low doses of coagulants (chitosan and PAC) cause formation of aggregates in cyanobacterial suspensions, but a ballast is needed to effectively sink the flocks out of the water column. Moreover, the efficient cyanobacteria removal by combining low dose of coagulant and ballast took place without great modification of cell surface charge (zeta potential). Those results are in good agreement with other studies that also have shown that the combination of a low dose of coagulant and a ballast is efficient in removing cyanobacteria from the water column [27, 28, 46]. Also in marine environments, combination of clay and coagulants may promote the sinking of harmful algae, where clays may act as ballast [21, 47, 48]. Furthermore, the use of PAC in addition to clay may improve the removal of extra-cellular harmful algal toxins from the water [49].

The ballast is added first followed immediately by the coagulant, which allows ballast particles to be entrapped inside the formed flocks. The low dose of chitosan and PAC used here (2 mg L^-1^) is similar to those used in other studies [20, 46, 50]. Such low dose of coagulant will not immediately damage the cells [51] and thus the combined low dose coagulant/ballast treatment leaves cells intact and precipitates them out of the water column. In field situations, the entrapped cyanobacteria will accumulate on the sediment where, after some days, coagulant induced lysis will occur [51, 52]. Released cyanotoxins can be degraded on the sediment as usually a wealthy community of cyanotoxin-degrading bacteria is present in those cyanobacteria infested lake sediments [23, 24]. A low dose of coagulant is essential to prevent cell lysis during the settling process and liberation of cell compounds, such as intracellular toxins into the water column, which is a common side effect of metal based algaecides [14, 16, 18] and hydrogen peroxide [18, 19]. Hence, combining a low dose coagulant with a ballast is a far more promising and safe curative measure than using algaecides.

Our observations that excellent flocculation and removal of cyanobacteria can be achieved without effects on the zeta potential when low dose of flocculants and a ballast are used, seems contradictory to a plethora of studies that have reported less negative zeta potentials are critical for attractive van der Waals forces to overcome the repulsive electrostatic forces of negative zeta potentials and thus to promote particles aggregation [25, 26, 53–55]. However, the charge neutralization or the electrical double layer compression are not the only forces in flocculation, but also inter-particle bridging can promote strong flocculation even when the zeta potential is low [56]. In addition to bridging, other flocculation mechanism that does not involve changes in charge and plays a role here is sweeping. In this process the cells are entrapped by large flocks formed due to polymeric precipitation or the formation of insoluble large metal hydroxide [57]. We postulate that the variety of monomeric and polymeric Al complexes [58] possibly formed in our experiments around the neutral pH values led to inter-particle bridging and not necessarily the impairment of the zeta potential. Likewise, for chitosan at circumneutral pH, bridging would be the main mechanism in flocculation [59]. Similar observations have been made for chitosan and kaolinite/bentonite where inter-particle bridging made neutralizing the surface charge of particles unnecessary [60]. At neutral pH and without major effects on the low negative zeta potentials, low doses of chitosan (1–2 mg L^-1^) caused strong water clearing due to bridging flocculation [60].

Our results apparently contradict the postulated importance of changing the zeta potential in order to remove cyanobacteria from the water column [25]. However, this happened because the flocculation took place at concentrations below the range of zeta potential driven aggregation. Moreover with regard to removal of cyanobacteria, such absence of strong effects on particle surface charges may be viewed as positive; interactions of cationic NH_3_^+^ groups of chitosan with the phosphoryl groups of phospholipid components of negative charged bacterial cell membranes increased membrane permeability and lysis [36, 37]. This led to release of cell contents [37]. Likewise, a high dose of alum (48 mg Al L^-1^) led to cell lysis and release of unwanted cell contents [61]. Hence, elevating the zeta potential by using higher amount of coagulants is not only unnecessary when the coagulant is combined with a ballast, but also undesirable given the potential risky side effects, as the release of cyanotoxins.

Considering ballasts, our experiments showed that combining a low dose of coagulant (either PAC or chitosan) with considerable proportions of silt and clay (above 200 mg L^-1^) seems suitable for precipitating cyanobacterial biomass independently of kind of the ballast used. On the other hand, the application of solely a ballast did not sink the cyanobacteria efficiently. The choice for the most appropriate ballast will follow from a system analysis. More expensive solid phase P sorbents, such as AMZ or LMB, will only be applicable in water bodies where the internal load is the major driver of cyanobacterial blooms, or where diffuse groundwater inflow is the major source of nutrients fuelling cyanobacterial blooms. In open systems, with continual nutrient inputs, cheaper, easy accessible ballast compounds will be far more suited.

In our experiments a ballast dose ranging from 25 to 400 mg L^-1^ was used. The latter dose might be viewed as unrealistically high implying high transport and application costs. However, it should be noted that the effective dose per water body needs to be determined running adequate flock and sink assays, and that usually the majority of the bloom is in the upper water layers. Once the upper water layer created cyanobacteria-ballast flocks sink they will further strip the underlying water column from particles. This means that the effective dose only needs to be calculated for the upper water layers and not for the entire lake volume, which will strongly reduce the amount of ballast needed. In Lake Rauwbraken (The Netherlands), for example, 77 mg L^-1^ LMB based on only the first meter of water was needed to precipitate positively buoyant *Aphanizomenon* from the upper water layer, while considering the entire water volume of the lake this dose was less than 10 mg L^-1^ LMB [39].

An important factor determining the dose of ballast is the density of the cyanobacterial bloom. Although many aspects of clay dispersion and coagulant modified soil techniques to mitigate harmful phytoplankton blooms have been addressed [33, 46, 62], the role of bloom density has virtually been neglected. In heavy bloom conditions, more ballast will be needed to settle the cyanobacteria effectively out of the water column. Large aggregated positively buoyant cyanobacteria flocks will migrate upwards when the ballast dose is too low. This could clearly be seen in the treatment with 2 mg L^-1^ chitosan and 25 mg L^-1^ LRS (see Fig 1C) where 11% more cyanobacteria accumulated at the surface than in the control without coagulant and ballast. Hence, dose estimations should be performed closely prior to *in situ* application.

In summary, our results showed that combining a low dose coagulant with a ballast compound removed positively buoyant cyanobacteria effectively from the water column and settled them to the bottom. A low dose coagulant is sufficient to promote formation of flocks without influencing the zeta potential, while entrapment of ballast particles ensures the settling. Although local soils and (modified) clays are suitable as ballast, the dose of ballast is dependent on the cyanobacterial bloom density, where higher ballast dose is needed for heavier blooms. Thus, the combined coagulant-ballast technique provides a promising alternative to algaecides in open systems.

## Supporting information

S1 Dataset Data base of the experimens of “Flock & Sink” testing 1) the range of LRS (0–400 mg L^-1^) combined with chitosan (2 mg L^-1^) or PAC (2 mg Al L^-1^); 2) the differents ballast compounds; and 3) the cyanobacterial biomass. (XLSX)
